# Hospital acquired vancomycin resistant enterococci in surgical intensive care patients – a prospective longitudinal study

**DOI:** 10.1186/s13756-018-0394-1

**Published:** 2018-08-23

**Authors:** Stefanie Kampmeier, Annelene Kossow, Larissa Monika Clausen, Dennis Knaack, Christian Ertmer, Antje Gottschalk, Hendrik Freise, Alexander Mellmann

**Affiliations:** 1 Institute of Hygiene, University Hospital Münster, Robert-Koch-Strasse 41, 48149 Münster, Germany; 2 Institute of Medical Microbiology, University Hospital Münster, Münster, Germany; 3 Department of Anaesthesiology, Intensive Care and Pain Medicine, University Hospital Münster, Münster, Germany

**Keywords:** Vancomycin resistant enterococci, Hospital-acquisition, Risk factors, Surgical intensive care patients

## Abstract

**Background:**

Vancomycin resistant enterococci (VRE) occur with enhanced frequency in hospitalised patients. This study elucidates the prevalence of VRE on admission among surgical intensive care unit (SICU) patients, whether these patients are at special risk for VRE acquisition and which risk factors support this process.

**Methods:**

Patients admitted to SICUs of the University Hospital Münster were examined during August–October 2017. VRE screening was performed within 48 h after admission and directly prior to discharge of patients. In parallel risk factors were recorded to estimate their effect on VRE acquisition during SICU stay.

**Results:**

In total, 374 patients (68% male) with a median age of 66 years were admitted to one of the SICUs during the investigation period. Of all, 336 patients (89.8%) were screened on admission and 268 (71.7%) on discharge. Nine patients were admitted with previously known VRE colonisation. Twelve (3.6%) further patients were VRE positive on admission. During ICU stay, eight (3.0%) additional patients turned out to be VRE colonised. Risk factors found to be significantly associated with VRE acquisition were median length of stay on the ICU (14 vs. 3 days; *p* = 0.01), long-term dialysis (12.5% vs. 2.0% of patients; *p* = 0.05), and antibiotic treatment with flucloxacillin (28.6% vs. 7.2% of patients; *p* = 0.01) or piperacillin/tazobactam (57.1% vs. 26.6% of patients; *p* = 0.01).

**Conclusions:**

SICU patients are not at special risk for VRE acquisition. Previous stay on a SICU should therefore not be considered as specific risk factor for VRE colonisation.

## Background

Enterococci are an emerging pathogen in hospitalised patients [1]. These pathogens ubiquitously occur in the hospital environment and show a high tenacity on inanimate surfaces [2–4]. As a result, enterococcal infections emerge with a rising frequency. Additionally, enterococci have the ability of acquiring resistances to multiple antimicrobial agents and the capacity to transfer resistances to other pathogens via mobile genetic elements [5–7]. For this reason the prevalence of vancomycin resistant enterococci (VRE) has increased intensively [1]. Vancomycin resistance is associated with enhanced mortality, e.g. among patients with enterococcal blood stream infections [8]. Within hospital settings prevention of VRE transmission is therefore a major objective.

Infection control strategies to control VRE vary, depending on local guidelines. Usually bundle strategies are applied to prevent transmission of VRE between two patients [9]. These include contact precautions, intensified disinfection strategies, the usage of personal protective equipment and active surveillance [10, 11]. In this context, screening strategies are controversially discussed [12]. Both, generalised screening and a risk-adaptive screening, are possible approaches. Regarding the latter, the question arises, which patient groups should be included. Acquisition of VRE in critical ill patients has been associated with prolonged duration of hospital stay, previous hospitalisations, antibiotic treatment, long-term dialysis and immunosuppression [13–15]. Surgical intensive care patients host a variety of these risk factors, although not explicitly mentioned as risk clientele. Whether surgical intensive care patients per se are at a special risk to acquire VRE and should therefore be included in a risk adapted screening upon subsequent admission to a hospital has not yet been investigated. The present work addresses these questions and investigates risk factors making a VRE acquisition more probable.

## Methods

### Clinical setting and infection control measures

The 1500-bed University Hospital Münster comprises four interdisciplinary SICUs, hosting abdominal-, trauma-, neuro-, vascular- and thoracic-surgery patients. In total, capacity of all ICUs is 43 beds at its maximum distributed over 26 patient rooms.

Routinely, screening for multidrug-resistant organisms includes an admission-screening for methicillin-resistant *Staphylococcus aureus* (MRSA) and multidrug-resistant Gram-negative bacteria according to the national German guidelines is established [16, 17]. After coincidental detection of VRE in screening swabs and clinical samples of SICU patients, a prospective investigation was initiated, concentrating on VRE acquisition during a stay on a SICU.

Extended hygiene measures in case of VRE detection include contact isolation in a separate room; cohorting of multiple VRE patients is possible. Sanitary facilities are strictly separated and staff is instructed to wear personal protective equipment in case of entering a patient room, consisting of gloves, and gowns. Surface cleaning disinfection was performed once a day.

### Detection of risk factors for VRE acquisition

During the 2-month study period (August – October 2017), risk factors for VRE acquisition were prospectively recorded during the entire in-patient stay. These risk factors included demographic data (age, gender), the duration of stay on the respective SICU, underlying diseases (haemato-oncological, immunosuppressive diseases [e.g. autoimmune-disease, malignancies, HIV-infection, immunomodulatory drug treatment], hepatic insufficiency, liver transplantation, renal insufficiency, long-term dialysis) and medication (systemic glucocorticoid and antibiotic treatment) attributed to VRE acquisition and previous contacts to the healthcare system (admission from a foreign or domestic hospital, admission from an (in-house) ICU) [13–15, 18]. Patients with a pre-existing VRE status were not included in the analysis, in order to detect risk factors of VRE acquisition on the SICU ward.

### VRE screening, culture, antibiotic resistances, PCR testing methods

VRE screening was performed upon patients’ admission on the SICU as well as upon discharge of patients from the SICU in order to detect VRE acquisition during SICU stay. Hospital-acquired VRE was defined as acquisition > 48 h after hospitalisation on the SICU. All VRE not acquired during SICU stay were defined as pre-existing VRE. Swabs were obtained rectally (5 cm *ab ano*) (Transwab® m40 compliant, mwe, Corsham, Wiltshire, UK) and subsequently streaked onto chromogenic selective agar (VRESelect™, Biorad, Hercules, California, USA). Suspected colonies were confirmed via MALDI-TOF-MS (Bruker Corporation, Bremen, Germany). Susceptibility testing was performed in accordance with the current European Committee on Antimicrobial Susceptibility Testing (EUCAST) standards for clinical breakpoints (version 7.0) using the VITEK® 2 system (BioMérieux, Nürtingen, Germany). Vancomycin resistance was confirmed by the detection of *vanA*, *vanB*, *vanC1* and *vanC2/3* using the GenoType Enterococcus system (Hain Lifescience, Nehren, Germany). Additionally, subsequent whole genome sequence-based typing confirmed presence of *van*-genes in these isolates.

### Whole genome sequence-based typing

In order to elucidate the clonal relationship of VRE-strains, isolates were compared genetically via whole genome sequencing (WGS) using the Illumina MiSeq platform (Illumina Inc., San Diego, USA) and laboratory procedures as described previously [19]. Coding regions were compared in a gene-by-gene approach (core genome multilocus sequence typing, cgMLST) using the SeqSphere+ software version 4.1 (Ridom GmbH, Münster, Germany) [20]. To visualize the clonal relationship a minimum-spanning tree was generated using the same software. Genotypes that differed in ≤3 cgMLST targets were rated as closely related and highly suspected for a hospital-acquired transmission. For backwards compatibility with classical molecular typing, i. e. MLST, the MLST sequence types (ST) were extracted from the WGS data in silico.

### Statistical analysis

All data are expressed as absolute numbers or percentage, if not stated otherwise. Independent risk factors were determined in a two-step analysis. First, a univariate analysis was performed, selecting potential risk factors. Chi-Square test was used for categorical and the two-sided student’s t-test for comparison of numerical data. Second, a logistic regression was performed to ascertain the independency of risk factors. Statistical significance was declared at *p* ≤ 0.05.

## Results

### Screening adherence, acquired VRE, genotypes

Table 1 shows the detailed results of screening data. Of 374 admitted patients, 336 were screened on admission and 268 on discharge respectively, not considering patients with a confirmed VRE status prior to admission or discharge. In total eight hospital-acquired VRE could be detected. Of all pre-existing VRE (on admission), six presented a *vanA*, 14 a *vanB* and one both genotypes. Of all hospital-acquired VRE, one hosted a *vanA* and seven a *vanB* genotype.

**Table 1:** Screening data of SICU-admitted patients (*n* = 374) during August to October 2017

	Screening result				
	Positive			Negative	Total
	*vanA* genotype	*vanB* genotype	*vanA* and *vanB* genotype		
Pre-existing VRE	6 (66.7%)	3 (33.3%)	0 (0%)	/	9
Screening on admission	0 (0%)	11 (3.3%)	1 (0.3%)	324 (96.4%)	336
Screening on discharge	1 (0.4%)	7 (2.6%)	0 (0%)	260 (97.0%)	268

### Antimicrobial resistance expressions

In total, 29 VRE isolates of 26 patients (one patient was readmitted twice and one patient once) were tested for antimicrobial resistance expressions. Table 2 gives a detailed overview of tested substances.

**Table 2:** Collection dates, *van*-genotypes, MLST sequence types and antimicrobial resistance expression of VRE strains detected in SICU patients. Each row represents a single patient

Isolate no.	Collection date	Positive result	MLST ST	*Van* - genotype	AMP	SAM	AX	AMC	PRL	TPZ	IPM	CIP	LEV	VAN	TEC	QD	TGC	LNZ	CN-HLR	S-HLR
001 123	15/08/2017 01/09/2017	On admission Pre-existing	ST80 ST80	*vanB* *vanB*	r	r	r	r	r	r	r	r	r	r	s	s	s	r	–	–
021 119 143	07/07/2017 31/08/2017 04/09/2017	Pre-existing Pre-existing Pre-existing	ST721 ST721 ST721	*vanA* *vanA* *vanA*	r	r	r	r	r	r	r	r	r	r	r	s	s	s	+	–
025	02/07/2017	Pre-existing	ST117	*vanB*	r	r	r	r	r	r	r	r	r	r	s	s	s	s	–	–
076	25/08/2017	On admission	ST117	*vanB*	r	r	r	r	r	r	r	r	r	r	s	s	s	s	–	+
085	02/08/2017	Pre-existing	ST721	*vanA*	r	r	r	r	r	r	r	r	r	r	r	s	s	s	+	+
126	05/09/2017	On admission	ST117	*vanB*	r	r	r	r	r	r	r	r	r	r	s	s	s	s	–	–
189	12/09/2017	On admission	ST80	*vanB*	r	r	r	r	r	r	r	r	r	r	s	s	s	s	–	+
204	26/09/2017	On discharge	ST80	*vanB*	r	r	r	r	r	r	r	r	r	r	s	s	s	s	–	+
235	20/09/2017	On admission	ST117	*vanB*	r	r	r	r	r	r	r	r	r	r	s	s	s	s	–	–
248	27/03/2017	Pre-existing	ST721	*vanA*	r	r	r	r	r	r	r	r	r	r	r	s	s	s	+	–
251	22/09/2017	On admission	NS	*vanA + B*	r	r	r	r	r	r	r	r	r	r	r	r	s	s	+	+
256	22/09/2017	On admission	ST117	*vanB*	r	r	r	r	r	r	r	r	r	r	s	s	s	s	–	–
283	02/10/2017	On discharge	ST117	*vanB*	r	r	r	r	r	r	r	r	r	r	s	s	s	s	–	–
291	05/10/2017	On discharge	ST80	*vanB*	r	r	r	r	r	r	r	r	r	r	s	s	s	s	–	–
300	04/10/2017	On discharge	ST80	*vanA*	r	r	r	r	r	r	r	r	r	r	r	r	s	s	–	+
302	25/10/2017	On discharge	ST117	*vanB*	r	r	r	r	r	r	r	r	r	r	s	s	s	s	–	–
314	13/10/2017	On discharge	ST117	*vanB*	r	r	r	r	r	r	r	r	r	r	s	s	s	s	–	–
330	06/10/2017	On admission	ST117	*vanB*	r	r	r	r	r	r	r	r	r	r	s	s	s	s	–	+
335	04/10/2017	Pre-existing	ST80	*vanB*	r	r	r	r	r	r	r	r	r	r	s	s	s	s	–	–
338	09/10/2017	On admission	ST117	*vanB*	r	r	r	r	r	r	r	r	r	r	s	s	s	s	–	–
348	11/10/2017	On admission	ST117	*vanB*	r	r	r	r	r	r	r	r	r	r	s	r	s	s	–	+
351	11/10/2017	On admission	ST117	*vanB*	r	r	r	r	r	r	r	r	r	r	s	s	s	s	–	–
352	02/04/2013	Pre-existing	ST117	*vanA*	r	r	r	r	r	r	r	r	r	r	r	r	s	s	+	+
361	22/10/2017	On discharge	ST117	*vanB*	r	r	r	r	r	r	r	r	r	r	s	s	s	s	–	–
366	13/10/2017	On admission	ST117	*vanB*	r	r	r	r	r	r	r	r	r	r	s	s	s	s	–	–
372	30/10/2017	On discharge	ST117	*vanB*	r	r	r	r	r	r	r	r	r	r	s	s	s	s	–	–

*MLST* Multilocus sequence typing; *AMP* Ampicillin, *SAM* Ampicillin/sulbactam, *AX* Amoxicillin, *AMC* Amoxicillin/clavulanic acid, *PRL* Piperacillin, *TPZ* Piperacillin/tazobactam, *IPM* Imipenem, *CIP* Ciprofloxacin, *LEV* Levofloxacin, *VAN* Vancomycin, *TEC* Teicoplanin, *QD* Quinopristin/dalfopristin, *TGC* Tigecyclin, *LNZ* Linezolid, *CN-HLR* Gentamicin-high level resistance, *S-HLR* Streptomycin-high level resistance; *r* Resistant, *s* Susceptible, + − positive, − - negative, *NS* not sequenced

### Whole genome sequence-based typing and VRE transmission

**Fig. 1:**
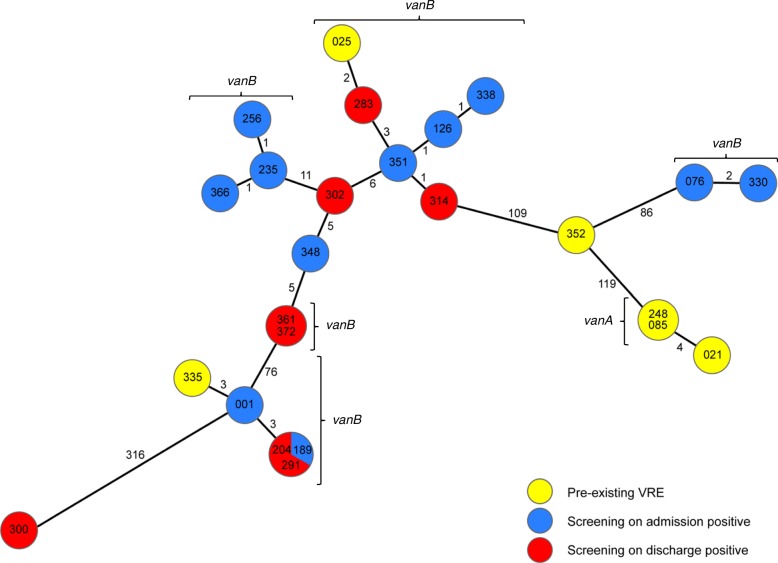
Minimum spanning tree of VRE isolates illustrating their genotypic relationship. Minimum spanning tree of 25 VRE strains isolated from intensive care unit patients with VRE anamnesis (*yellow*) and detected during screening on admission (*blue*) and on discharge (*red*) during August and October 2017 based on 1423 cgMLST target genes [20], pairwise ignoring missing values. Genotypes are numbered chronologically in order of patients’ admission on ICU. Each dot represents one genotype. Size of dots correlates with the number of identical genotypes. Numbers near to the connecting lines show the number of alleles differing between two genotypes. Whole Genome Sequencing revealed six clusters of VRE, one *vanA*-cluster and five *vanB*-clusters

**Fig. 2:**
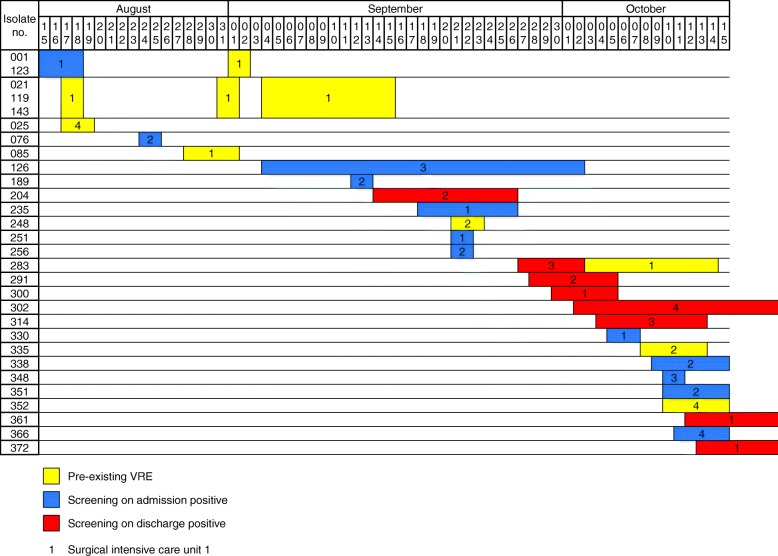
Timeline of detected VRE isolates illustrating time overlap. Timeline of 29 VRE strains isolated from intensive care unit patients with VRE anamnesis (*yellow*) and detected during screening on admission (*blue*) and on discharge (*red*) during August and October 2017. Each row represents one patient. Numbers within coloured boxes indicate the surgical intensive care unit the patient was admitted to. Isolates 302, 361 and 372, detected in screening on discharge, were identified after end the of study period, but included, as the patients, strains were isolated from, were admitted during the observation period

Of all 29 isolates 25 were subjected to whole genome sequencing. In patients with repetitive VRE detections and the same antimicrobial resistance expression, only the first isolate was undertaken the sequencing procedure. One isolate was not sequenced. Analysing the MLST ST of all sequenced isolates, ST117 was most prevalent (64.0%) followed by ST80 (24.0%) and ST721 (11.0%) (see also Table 2). A genetical comparison with the help of the cgMLST scheme, based on 1423 genes present in all isolates, revealed six clusters, comprising two, three, five and six isolates respectively (Fig. 1). Two of these clusters contain genotypes of pre-existing isolates and isolates detected in both screenings. Six out of eight VRE isolates (204, 283, 291, 314, 361, 372) detected in patients on discharge, who were previously tested negative for VRE on admission, are genetically closely related to isolates detected in other patients, suggesting a transmission of one VRE clone on ward in these cases (see also Fig. 2). The other two isolates (300, 302) detected on discharge are genetically unrelated to other isolates, which can be attributed to selection of VRE e.g. via antibiotic application in these patients or a false-negative screening result on admission.

### Analysis of risk factors in SICU patients

Risk factors were compared between the group of hospital-acquired VRE-patients and all patients being not VRE-colonised or infected hospital-acquired. Patients with a pre-existing VRE status were not included in the analysis, in order to detect risk factors of VRE acquisition on the SICU ward. Risk factors to have a significant influence on VRE acquisition in this patient clientele were found to be long-term dialysis (*p* = 0.05), the median duration of stay (*p* = 0.01), and an antibiotic treatment with flucloxacillin (duration of treatment for 5.3 days [*p* = 0.01]) or piperacillin/tazobactam (duration of treatment for 4.6 days [*p* = 0.01]), while an antibiotic treatment per se did not show a significant risk profile. Further results of the risk factor analyses can be found in Table 3. The multivariate analysis found the duration of stay and flucloxacillin treatment to be statistically significant as shown in Table 4.

**Table 3:** Characteristics and risk factors of admitted surgical intensive care patients with and without hospital-acquired VRE

Characteristic	All admitted patients (n = 374)	Patients with acquired VRE (*n* = 8)	Patients without acquired/ pre-existing VRE (*n* = 345)	*p*-value
Demographic data				
Median age (years)	66 (range: 14–91)	71.5 (range: 50–78)	65 (range: 14–91)	0.43
Male gender	254 (67.9%)	4 (50.0%)	241 (69.9%)	0.23
Median duration of stay (days)	3 (range: 1–45)	14 (range: 7–30)	3 (range: 1–45)	*0.01*
Underlying disease/treatment				
Haemato-oncological disease	62 (16.6%)	1 (12.5%)	58 (16.8%)	0.74
Immunosuppressive disease	71 (19.0%)	1 (12.5%)	66 (19.1%)	0.63
Hepatic insufficiency	17 (4.5%)	0 (0%)	15 (4.3%)	0.55
Liver transplantation	8 (2.1%)	0 (0%)	8 (2.3%)	0.66
Renal insufficiency	51 (13.6%)	2 (25.0%)	41 (11.9%)	0.26
Long-term dialysis	10 (2.7%)	1 (12.5%)	7 (2.0%)	*0.05*
Systemic glucocorticoid treatment	36 (9.6%)	1 (12.5%)	32 (9.2%)	0.76
Antibiotic treatment	218 (58.3%)	7 (87.5%)	207 (60.0%)	0.11
Ampicillin	7 (3.2%)	0 (0%)	4 (1.9%)	0.76
Amoxicillin	14 (6.4%)	0 (0%)	13 (6.3%)	0.58
Flucloxacillin	20 (9.2%)	2 (28.6%)	15 (7.2%)	*0.01*
Piperacillin/tazobactam	62 (28.4%)	4 (57.1%)	55 (26.6%)	*0.01*
Cefuroxime	130 (59.6%)	1 (14.2%)	127 (61.4%)	0.16
Ceftriaxone	7 (3.2%)	0 (0%)	3 (1.4%)	0.79
Meropenem	41 (18.8%)	2 (28.6%)	36 (17.4%)	0.19
Clindamycin	7 (3.2%)	0 (0%)	7 (3.4%)	0.68
Daptomycin	8 (3.7%)	0 (0%)	5 (2.4%)	0.73
Linezolid	3 (1.4%)	0 (0%)	3 (1.4%)	0.79
Rifampicin	13 (6.0%)	0 (0%)	10 (4.8%)	0.63
Erythromycin	7 (3.2%)	0 (0%)	6 (2.9%)	0.71
Vancomycin	19 (8.7%)	0 (0%)	16 (7.7%)	0.53
Fosfomycin	7 (3.2%)	0 (0%)	6 (2.9%)	0.71
Trimethoprim/Sulfamethoxazole	6 (2.8%)	0 (0%)	6 (2.9%)	0.71
Metronidazole	8 (3.7%)	0 (0%)	7 (3.4%)	0.68
Previous contact to healthcare system				
Admission from a foreign hospital	1 (0.3%)	0 (0%)	1 (0.3%)	0.88
Admission from a domestic hospital	188 (50.2%)	5 (62.5%)	173 (50.0%)	0.49
Admission from an intensive care unit	90 (24.1%)	4 (50.0%)	76 (22.0%)	0.06

Statistical significance was declared at *p* ≤ 0.05 (see italicized entries)

**Table 4:** Multivariate analysis: risk factors associated with VRE acquisition

Risk factors (*p* ≤ 0.05)	Odds Ratio	95% CI
Duration of stay	0.90	0.84–0.96
Long-term-dialysis	0.08	0.01–1.10
Flucloxacillin treatment	0.09	0.01–0.60
Piperacillin/tazobactam treatment	0.24	0.05–1.13

## Discussion

Control of VRE is an emerging topic, patients and healthcare workers have to cope with. An important aspect for prevention of transmission is the early recognition of VRE via screening strategies. Here, the question was addressed, whether SICU patients acquire VRE during their stay and which factors put them at special risk for acquisition. VRE acquisition rate (3.0%) on the SICU was lower than the initial prevalence (3.6%) on admission of all patients. Additionally, no hospital-acquired infection but only colonisations occurred. Distribution of detected *van*-genotypes and MLST ST (*vanB*-genotype and ST117 were most prevalent) are thereby in accordance with national results and trends published for German healthcare institutions recently [21]. In contrast to another retrospective study investigating hospital-acquired VRE, where the acquisition rate was 28.6%, on different wards including surgical and internal ICUs and lacking a generalised VRE screening, acquisition of VRE in our investigation was comparably low [22]. Further investigations on haemato-oncological patients revealed one third of these patients to develop hospital-acquired VRE [18]. SICU patients should per se not be considered as a specific risk clientele for VRE acquisition in comparison with these patients.

Nevertheless, since hospital-acquired VRE colonisations occurred in our patients, risk factors supporting a VRE acquisition in this group of patients were assessed. Here, the median duration of stay, long-term dialysis and the antibiotic treatment with flucloxacillin or piperacillin/tazobactam were found to be the predominant risk factors. These data correspond with previously investigated risk factors and certainly cannot be considered independently but presuppose each other [23, 24], as also verified by the multivariate analysis. Other risk factors that were found to play an important role in VRE acquisition in other patient clientele could not be verified for our SICU patients. Here, the attributed risk of VRE acquisition after cephalosporin treatment needs to be mentioned. Due to perioperative antibiotic prophylaxis standards, especially referring to cardiac surgery patients, approximately 60% of all admitted patients received cefuroxime; however, this did not lead to an increased VRE acquisition rate. This is noteworthy, since cephalosporine use promotes selection of enterococci and thereby VRE due to intrinsic resistance mechanisms. Nevertheless, previous studies could confirm, that cephalosporine use per se during hospitalization does not result in an enhanced VRE carriage [14]. Interestingly, since the duration of application of flucloxacillin and piperacillin/tazobactam did not vary significantly among the hospital-acquired VRE and the non VRE group, the present results suggest that few antibiotic applications can be sufficient to potentially select VRE. The impact of relatively low numbers of antibiotic administrations on selection of certain pathogens corroborates similar findings for the selection of *Clostridium difficile* [25].

Previous contacts to VRE confirmed patients are often mentioned as a major risk factor in VRE acquisition [26]. Considering the present core genome analysis of isolates, transmission of VRE on ward could not explicitly be excluded in some cases. This may be due to the transmission of this pathogen on inanimate surfaces in advance to the final diagnosis of the VRE status. Hence in case of a confirmed VRE status, adequate basic and, if necessary, intensified hygienic measures consisting of bundle strategies including contact precautions, usage of personal protective equipment, appropriate disinfection strategies, screening of contact patients and antibiotic stewardship should be implemented to avoid transmissions as good as possible.

Our study has limitations. First, the study period was relatively short. Additional risk factors might have been revealed in case of a longer study period. Moreover, distribution of detected MLST ST in found VRE could have been different. Second, we evaluated risk factors promoting VRE carriage, e.g. flucloxacillin and piperacillin/tazobactam application, but did not investigate the pathophysiological background. Future (prospective) studies are needed to reveal underlying mechanisms. Nevertheless, our findings are in accordance with results published previously [27]. Environmental sampling was not performed during the present study, which could have uncovered the role of inanimate surfaces in VRE transmission, especially in cases of clonality of isolates. Another limitation is the problem of screening sensitivity. Here, only one rectal swab was applied on admission and on discharge respectively, which can lead underestimation of VRE prevalence. However, since screening was performed the same way on admission and on discharge, acquisition rate of VRE during SICU stay can still be assessed precisely.

## Conclusion

Compared to patient clienteles previously investigated [23, 28–30], SICU patients per se are not at higher risk of VRE acquisition if hygiene measures are applied as recommended. If a risk adapted screening policy is applied as practised in other national institutions [22], stay on a SICU should not be considered as a risk factor for screening upon admission to a healthcare facility.

## Abbreviations

SICU: Surgical intensive care unit
VRE: Vancomycin resistant enterococci
